# Uncertainties associated with assessing the public health risk from *Legionella*

**DOI:** 10.3389/fmicb.2014.00501

**Published:** 2014-09-24

**Authors:** Harriet Whiley, Alexandra Keegan, Howard Fallowfield, Kirstin Ross

**Affiliations:** ^1^Health and the Environment, Flinders UniversityAdelaide, SA, Australia; ^2^South Australian Water CorporationAdelaide, SA, Australia

**Keywords:** *Legionella*, L. pneumophila, risk assessment, QMRA, Legionellosis, public health

## Abstract

*Legionella* is an opportunistic pathogen of public health concern. Current regulatory and management guidelines for the control of this organism are informed by risk assessments. However, there are many unanswered questions and uncertainties regarding *Legionella* epidemiology, strain infectivity, infectious dose, and detection methods. This review follows the EnHealth Risk Assessment Framework, to examine the current information available regarding *Legionella* risk and discuss the uncertainties and assumptions. This review can be used as a tool for understanding the uncertainties associated with *Legionella* risk assessment. It also serves to highlight the areas of *Legionella* research that require future focus. Improvement of these uncertainties will provide information to enhance risk management practices for *Legionella,* potentially improving public health protection and reducing the economic costs by streamlining current management practices.

## INTRODUCTION

**FIGURE 1:**
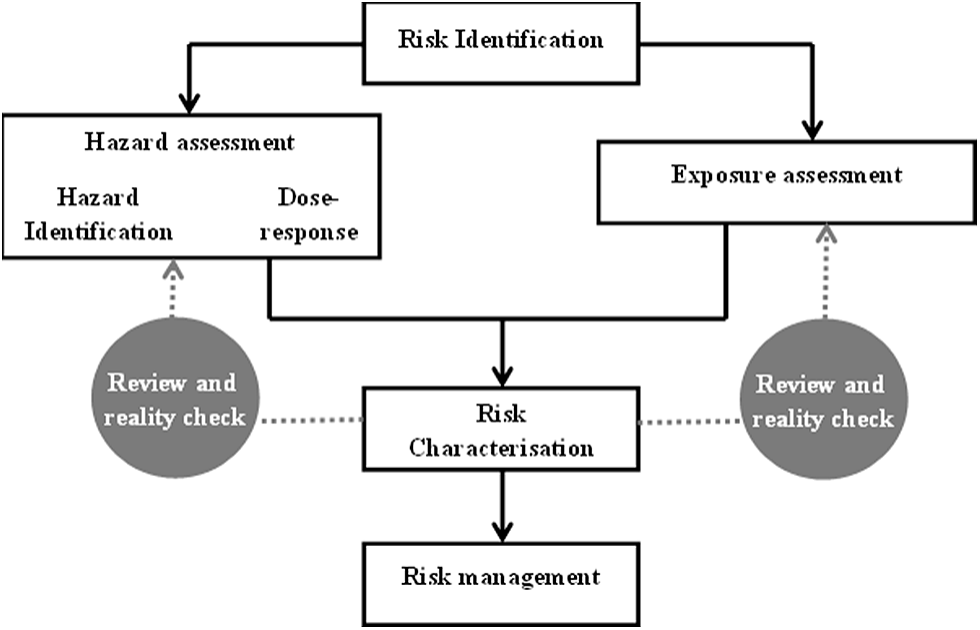
**EnHealth risk assessment framework adapted from Priestly et al. (2012)**.

**FIGURE 2:**
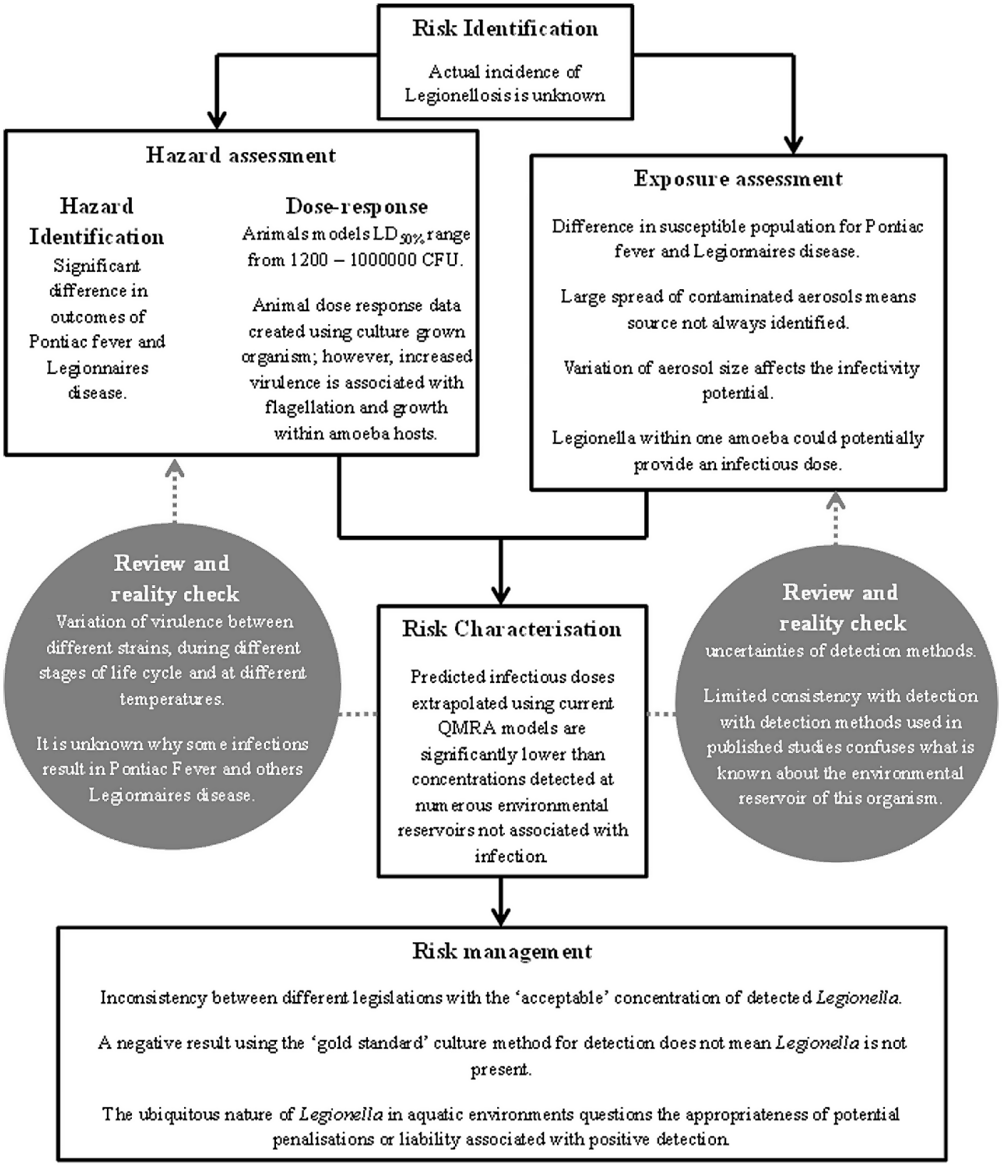
**Uncertainties of *Legionella* risk assessment highlighted through each step of the EnHealth risk assessment frame work**.

*Legionella* spp. is the causative agent of Legionellosis and has been identified as a public health concern since 1976 (Fields et al., 2002; Bartram et al., 2007; Berger, 2012). Currently, government bodies rely on risk assessment models to inform the development of regulatory tools for the control of Legionellosis (Cooper et al., 2004). Current *Legionella* risk assessments may be compromised by uncertainties in *Legionella* detection methods, strain infectivity and infectious dose. This paper follows the EnHealth Risk Assessment Framework (**Figure 1**) developed in Australia to review current knowledge of *Legionella* risk and discuss the uncertainties and assumptions made. The EnHealth risk assessment framework was adopted by the Australian government to provide a national approach for assessing human health risks from environmental hazards. It provides a benchmark for risk assessments that are being undertaken for a wide variety of projects by governments and industry in Australia (Priestly et al., 2012). The uncertainties associated with each component of the risk assessment framework are collated in **Figure 2** and provide a useful tool when evaluating data used for *Legionella* risk assessment.

## RISK IDENTIFICATION

Worldwide, *Legionella pneumophila* is the most common causative agent of Legionellosis (Buchbinder et al., 2002). Recently, a global increase in the incidence of reported Legionellosis has been observed (Centers for Disease Control and Prevention, 2011; Beauté et al., 2013). In 2011, there were 4897 confirmed Legionellosis cases across Europe (incidence rate of 0.97 cases per 100,000; European Centre for Disease Prevention and Control, 2013). and 4,202 cases across the United States (incidence rate of 1.36 cases per 100,000; Centers for Disease Control and Prevention, 2013a). In 2013, Australia recorded 2.2 cases of Legionellosis per 100,000 (Department of Health, 2014). The true incidence of Legionellosis may be much higher as many community acquired cases go unreported (Marston et al., 1997; Todd, 2005).

Legionellosis outbreaks are primarily associated with artificial aquatic environments (Fields et al., 2002). Hence, the risk assessment for *Legionella* is especially important for public health officials and managers responsible for maintenance of water distribution systems and cooling towers within industrial or public buildings (Cooper et al., 2004). Risk identification is the first component of the risk assessment framework, for *Legionella* this is limited as the true incidence of Legionellosis is unknown and it has been estimated that the true incidence of Legionellosis could be 20 times greater than the currently reported incidence (Marston et al., 1997). Many Legionellosis community acquired cases go unreported, which places the focus of nosocomial infection and makes assumptions regarding disease epidemiology within the whole community difficult.

## HAZARD ASSESSMENT

Legionellosis collectively refers to clinical syndromes as a consequence of *Legionella* infection (Fields et al., 2002). This includes Pontiac fever, a self-limiting febrile illness and Legionnaires diseases, a severe multisystem illness involving atypical pneumonia (Buchbinder et al., 2002; Fields et al., 2002; Bartram et al., 2007). The mortality rates of Legionellosis are highly variable and can range from 1 to 80%, depending on the underlying health of a patient, promptness of diagnosis and treatment and whether the disease is nosocomial, sporadic or part of an outbreak (Bartram et al., 2007; Diederen, 2008). Currently, there is no consensus as to why exposure to *L. pneumophila* may result in either Pontiac fever or Legionnaires disease (Diederen, 2008; Remen et al., 2011). Remen et al. (2011) identified cases of Pontiac fever from 104 nurses working at 19 different retirement homes over a 4 month period and found no association with concentrations of *Legionella* detected from the retirement village showers and cases of Pontiac fever. Occasionally simultaneous outbreaks of Pontiac fever and Legionnaires’ disease from the same source have been observed (Bartram et al., 2007; Euser et al., 2010). A greater understanding of the epidemiology of these vastly different clinical outcomes would significantly improve *Legionella* risk assessment models. In 2007 the overall case fatality rate for reported cases of Legionellosis across Europe was 6.6% (Joseph and Ricketts, 2010) and from 2005 to 2009 the case fatality rate was 8% across the United States (Centers for Disease Control and Prevention, 2011). The annual cost of hospitalisations due to Legionellosis in the United States is estimated to exceed US$716 million (Giambrone, 2013).

There are limited data regarding human dose response for *L. pneumophila* and the concentration of *Legionella* required to result in an outbreak is unknown (O’Brien and Bhopal, 1993; Armstrong and Haas, 2007a). The organism is ubiquitous to many natural and artificial environments which suggest people are frequently exposed to low concentration of the organism with no consequence or asymptomatic production of *Legionella* antibodies (Bartram et al., 2007). This was demonstrated by Boshuizen et al. (2001) who investigated an outbreak of Legionnaires disease caused by a display whirlpool spa at a floral trade show and found that 742 exhibitors without Legionnaires disease had higher average antibody levels than the general population. The exhibitors were surveyed regarding their whereabouts during the fair and those who ventured closer to the whirlpool spa had higher antibody levels. The data from animal models for *Legionella* dose response have been used for quantitative microbial risk assessment (QMRA) purposes. *In vitro* inhalation exposure data for *L. pneumophila* is available for guinea pigs (Davis et al., 1982; Breiman and Horwitz, 1987), mice (Wright et al., 2003), rats (Davis et al., 1982), marmosets (Baskerville et al., 1983), and monkeys (Kishimoto et al., 1979; Baskerville et al., 1983). However, the infectious dose (LD_50%_) across these animal models range from 1200 to 1000000 CFU (colony forming unit; Armstrong and Haas, 2007a). Guinea pigs models have been generally accepted as the most appropriate representation of human dose response for *L. pneumophila,* primarily because *in vitro* studies show similarities for *Legionella* uptake, survival and replication within guinea pigs and human macrophages (Rechnitzer et al., 1992; Armstrong and Haas, 2008). Armstrong and Haas (2007b) used guinea pig ID_50%_ (12 CFU) to a to create a QMRA model for *Legionella* exposure (Armstrong and Haas, 2007a), the justification for using this guinea pig model was also published (Armstrong and Haas, 2008). This study used composite data from animal dose response models, average environmental concentrations from previous studies and exposure data from three outbreaks, one associated with one whirlpool spa and two hot spring spas. From this QMRA model the predicted infectious dose from the whirlpool spa was a mean of 10 CFU and had a 95% range of 1.3–34 CFU, and the predicted infectious dose for the two hot spring spas was a mean of 47 CFU with a 95% range of 24–84 and for the other a mean of 2.3 CFU with a 95% range of 1.1–4.1 CFU. Although the models acknowledges uncertainties associated with the QMRA model, the final predicted infectious dose values calculated for the specific outbreaks are significantly lower compared to the concentrations of *Legionella* detected from environmental sources not associated with infection reported in numerous published studies (Buchbinder et al., 2002; Valster et al., 2011; Wang et al., 2012). The limitations of data used for the *Legionella* qMRA model were acknowledged by Armstrong and Haas (2007a). Improvements of this model can only be achieved through future research and greater understanding of *Legionella* epidemiology.

Uncertainties with *Legionella* dose response data also arise due to the large variation in virulence of environmental *Legionella* strains (Bollin et al., 1985b; Alli et al., 2003). Several studies have demonstrated that variation in growth temperature affect the virulence of *L. pneumophila* (Edelstein et al., 1987; Mauchline et al., 1994). However, even these studies are conflicting, Edelstein et al. (1987) reported *L. pneumophila* grown at 25°C were more virulent compared to those grown at 41°C; whereas Mauchline et al. (1994) reported that *L. pneumophila* grown at 37°C were more virulent than those grown at 24°C. Increased virulence of *L. pneumophila* is also associated with flagellation which is life cycle dependent and genetically associated to the expression of a virulent phenotype (Heuner and Steinert, 2003). Cirillo et al. (1999) also reported that *L. pneumophila* grown intracellular within an amoeba host has greater virulence than culture grown strains.

The disparity between Legionnaires disease and Pontiac fever further confounds *L. pneumophila* infectious dose data. Currently there is no consensus for an epidemiological definition of Pontiac fever (Tossa et al., 2006). Furthermore, some experts believe that Pontiac fever is caused by exposure to a mixture of live and dead microorganisms including endotoxins made by non-*Legionella* bacteria plus low doses of live or dead *Legionella* which are unable to cause pneumonia in the infected host. However, more research is required to confirm this assumption (Burnsed et al., 2007; Edelstein, 2007; Diederen, 2008). Legionnaires disease and Pontiac fever vary in regards to patients risk factors and disease outcomes (Diederen, 2008). The incubation period for Legionnaires disease is 2–10 days (Bartram et al., 2007); whereas Pontiac fever has an incubation period of 30–90 h (Pancer and Stypukowska-Misiurewicz, 2002).

## EXPOSURE ASSESSMENT

Men aged 40 years and over with underlying health issues including smoking, alcohol abuse, diabetes, heart disease, and other immunosuppression are the most susceptible population for community acquired or travel associated Legionnaires disease. Susceptible patients for nosocomial Legionnaires disease include transplant recipients, other immunosuppression, surgery, cancer, diabetes, treatment with respiratory devices, chronic heart or lung disease, smoking and alcohol abuse, which are also associated with higher mortality rates (Fields et al., 2002; Bartram et al., 2007). However, Pontiac fever preferentially affects the younger population and the median age range from several outbreaks was reported to be 29–32 years (Tossa et al., 2006). Age, gender, and smoking have not been observed to be risk factors for Pontiac fever (Friedman et al., 1987).

*Legionella* is present in a range of aquatic environments and human infection occurs through the inhalation of contaminated aerosol or aspiration of contaminated water (Bartram et al., 2007). Incidences of Legionellosis have been linked to contaminated shower heads (Hanrahan et al., 1987; Zmirou-Navier et al., 2007), spas (Jernigan et al., 1996; Benkel et al., 2000), baths (Sasaki et al., 2008) a hospital steam towel warmer (Higa et al., 2012), ice machines (Graman et al., 1997; Schuetz et al., 2009), mist generators (Mahoney et al., 1992), decorative water fountains (Fleming et al., 2000; O’Loughlin et al., 2007; Haupt et al., 2012), hospital water distribution systems (Tobin et al., 1981; Hanrahan et al., 1987) dental units (Reinthaler et al., 1988; Atlas et al., 1995) and cooling towers (Isozumi et al., 2005; Nguyen et al., 2006). *L. pneumophila* has also been detected in potable water and in 2011, 57.6% of all potable water related disease outbreaks in the United States were due to *Legionella* spp. (Centers for Disease Control and Prevention, 2013b). A recent study also used quantitative polymerase chain reaction (qPCR) to detect *Legionella* spp and *L. pneumophila* ubiquitously through South Australian potable and reuse water distribution pipelines. Within the potable water distribution system *Legionella* spp and *L. pneumophila* was detected at maximum concentrations of 10^6^ and 10^3^ copies/mL respectively (Whiley et al., 2014). Human to human transmission of *Legionella* has not been observed (Albert-Weissenberger et al., 2007).

There have been numerous studies which have investigated the production, size and spread on *Legionella* contaminated aerosols (Bollin et al., 1985a; Ishimatsu et al., 2001; Nguyen et al., 2006; Dutil et al., 2007; Zmirou-Navier et al., 2007; Chang et al., 2010). The ability of *Legionella* to access the human respiratory tract is governed primarily by the size of the aerosol. Aerosols >10 μm in diameter get captured within the nose and throat, between 5 and 10 μm and aerosols can reach the upper and lower respiratory tract and between 2 and 5 μm they can reach the lungs and conducting airways (Cox and Wathes, 1995). In Bollin et al. (1985a) demonstrated that 90% showerhead aerosol contaminated with *L. pneumophila* sampled above a shower door were between 1 and 5 μm in diameter and 50% of *Legionella* contaminated aerosols from facets were 1–8 μm in diameter. These aerosols are small enough to efficiently transport the *L. pneumophila* into the lower respiratory system. The production of aerosols also provides *Legionella* a method to further spread contamination. This is particularly important for cooling towers. Nguyen et al. (2006) demonstrated that contaminated aerosols from a cooling tower identified as the source of an outbreak of legionnaires’ disease spread up to 6 km from the cooling tower. Dennis and Lee (1988) demonstrated that virulent strains of *L. pneumophila* survived longer within aerosols compared to avirulent strains, which is important to consider when determining the potential spread of contaminated aerosols.

This difference in susceptible population for Legionnaires disease and Pontiac fever is a significant limitation for *Legionella* risk assessment. The potential for contaminated aerosols to spread considerable distances makes it challenging to identify the origin of the aerosol and limits knowledge regarding sources of Legionellosis (Nguyen et al., 2006). Variation in the size of aerosols also affects the infectivity, which makes it difficult to determine the infectious dose and what environmental concentrations are considered acceptable.

In order to quantify the risk of Legionellosis, enumeration of *Legionella* from a source is required. Many regulatory guidelines are based on the detection of *Legionella.* For example, in Australia each state has different cooling tower legislation regarding *Legionella.* In South Australia, Queensland and Australian Capital Territory detection of ≥1000 *Legionella* CFU/mL from a cooling tower water sample requires mandatory reporting to the relevant health department (Australian Capital Territory Department of Health, 2005; Workplace Health and Safety Queensland, 2008; South Australian Department of Health and Aging, 2013). Whereas, in Victoria mandatory reporting is required if there are three consecutive detections of *Legionella* ≥10 CFU/mL (Department of Health, 2009). The problem with this legislation is the inherent difficulty regarding the detection of *Legionella* from environmental samples (Hussong et al., 1987; Centers for Disease Control and Prevention, 2005; Whiley and Taylor, 2014).

Currently, culture is considered the “gold standard” for *L. pneumophila* detection (Reischl et al., 2002). However, the slow growth rate of *L. pneumophila* makes the method tedious and can be inaccurate due to plate being overgrown from faster growing organism (Bopp et al., 1981; Hussong et al., 1987). Further inaccuracies occur with variation of sample holding time prior to culturing. McCoy et al. (2012) demonstrated that sample holding time significantly impacted *Legionella* recovery by culture, with enumerated *Legionella* changing by up to 50% within 6 h and up to 2 log_10_ difference after 24 h. In Australia the standard holding time for NATA (National Association of Testing Authorities) accredited laboratories is <8 h (McCoy et al., 2012). Inaccuracies with culture enumeration may also occur if final confirmation of all *Legionella* isolates are not performed using an alternative method such as 16s RNA sequencing, polymerase chain reaction (PCR), latex agglutination test, or immunofluorescence antibody test. Borges et al. (2012) used the standard *Legionella* culturing method and found that 40 isolates from natural and artificial water samples grew on GVPC selective *Legionella* agar, had the same morphological “ground glass” appearance of *Legionella*, and when restreaked onto blood agar isolates did not grow. However, 16s RNA sequencing confirmed that the isolates were not *Legionella* and in fact were from the *Chitinophagaceae* family. Although not an issue in accredited laboratories which would complete final confirmation tests, it does present the possibility of false positives when culturing *Legionella*, a concept that should be considered when reading past studies relying on culture for detection.

A significant limitation of culture detection is that it does not account for the presence of viable but non-culturable (VBNC) organisms (Chang et al., 2009). Studies have shown that *Legionella* becomes VBNC during starvation, when exposed to high temperatures and monochloramine disinfection (Chang et al., 2007; Alleron et al., 2008). Allegra et al. (2011), compared *Legionella* detected from hospital water systems using culture and a flow cytometry assay to identify VBNC cells and found that VBNC cells varied from 4.6 to 71.7%. The problem with the presence of VBNC *Legionella* is that using the viable culture method of detection a negative result does not necessarily mean that *Legionella* is not present. This has serious ramifications for public health protection using routine sampling.

*Legionella* detection using qPCR is becoming a popular alternative to culture methods as it has a quick turnaround time and high specificity. The main problem with qPCR is that it enumerates both live cells and intact killed cells (Delgado-Viscogliosi et al., 2009). This means there is a significant discrepancy between detection of *Legionella* using either culture or qPCR. A review of studies which detected *Legionella* from environmental samples with culture and qPCR simultaneously found that from a total of 28 studies, 2856/3967 (72%) samples tested positive for *Legionella* spp. using qPCR and 1331/3967 (34%) using culture (Whiley and Taylor, 2014). This discrepancy highlights the limitation of both the current detection methods and potential concerns with relying on these results for risk assessment purposes.

Another difficulty of detection from environmental sources is the ability of *Legionella* to opportunistically parasitise free living protozoa (Walser et al., 2014). Berk et al. (1998) demonstrated that vesicles expelled from amoeba may contain 20–200 *Legionella;* however, only one CFU was detected using culture. This study also demonstrated that over 90% of vesicles containing *L. pneumophila* expelled from *Acanthamoeba polyphaga* and *A. castellanii* were 2.1–6.4 μm in diameter which is within the respirable size range. A single *A. polyphaga* was able to expel 25 *L. pneumophila* filled vesicles over a 24 h period. Buse and Ashbolt (2012) demonstrated that under conditions representative of a drinking water system the maximum number of *L. pneumophila* release from *A. polyphaga* and *Naegleria fowleri* was respectively 1,348 and 385 CFU per trophozoite. Comparison of these concentrations to a guinea pig aerosol infection model (Berendt et al., 1980) demonstrated that as few as 1–75 infected amoebae within aerosols may contain enough pathogenic *L. pneumophila* to cause human infection.

The significant discrepancies between infectious dose models and detection methods has resulted in published studies giving *Legionella* counts which are potentially meaningless for risk assessment purposes. Armstrong and Haas (2007a) extrapolated animal modeling and data from 3 outbreaks of *Legionellosis* for QMRA modeling and predicted infectious doses or *Legionella* ranging from 1.3 to 47 CFU. The governments of South Australia, Queensland and Australian Capital Territory require mandatory reporting if *Legionella* is detected at a concentration ≥1000 *Legionella* CFU/mL from a cooling tower water (Australian Capital Territory Department of Health, 2005; Workplace Health and Safety Queensland, 2008; South Australian Department of Health and Aging, 2013). Wang et al. (2012) used qPCR to detect *Legionella* in potable water from point of use at maximum concentrations of 2.3 × 10^3^ ± 9 × 10^2^ copies/mL. Whiley et al. (2014) used qPCR to detect *Legionella* at a dead-end of a potable water distribution system at a maximum concentration of 10^6^ copies/mL. The inconsistencies of these values highlight the biggest flaw with current *Legionella* risk assessment and question the value of routine sampling. The discrepancies between *Legionella* concentrations measured using the different detection methods also make it difficult to compare findings from published studies. This makes it challenging to identify environmental sources of potential public health significance and to compare the effectiveness of different control measures and protocols.

## RISK CHARACTERIZATION

Presently there are risk assessments models available for *Legionella* (Bentham, 2003; Mouchtouri et al., 2010; Torrisi et al., 2012). This include QMRA models for *Legionella* exposure from spas (Armstrong and Haas, 2007b), distributed water (Storey et al., 2004), and rainwater (Ahmed et al., 2010). These risk assessments characterize the nature and magnitude of risk associated with environmental sources of Legionellosis using the information currently available. However, often results of these risk assessments are not consistent or considerate of the literature regarding *Legionella* in the environment and its ubiquitous nature in aquatic environments. When utilizing risk assessments for the purpose of regulatory tools the realities of the limitation and assumptions made must be taken into consideration, particularly when considering potential cases of liability.

## RISK COMMUNICATION AND MANAGEMENT

Currently in most developed countries there are many models of risk communication regarding Legionellosis including: training and education programs, management procedures and established documentation and communication procedures (Cooper et al., 2004; Bartram et al., 2007). Current risk management strategies for *Legionella* in built water systems are focused on maintaining overall system health to control biofilm formation. This can be achieved by maintaining water temperature at <20°C or >50°C, periodical flushing of the system with hot water, or disinfection with biocides, copper–silver ionization, anodic oxidation or ultra violet light (Sidari et al., 2004; Bartram et al., 2007). The uncertainties associated with *Legionella* risk assessment presented in this paper also highlight areas requiring greater research in the future.

Routine testing for *Legionella* is required by most regulatory bodies. This is aimed at monitoring the effectiveness of treatment and management protocols, but also is a result of political expediency. Politicians and government officials often require routing testing for *Legionella* to demonstrate that the public health risk is being managed, despite the uncertainties of current detection methods. The main danger of this is the false sense of security gained from a negative *Legionella* test result, as there is little correlation between a positive *Legionella* test results using culture and human health risks (Kool et al., 1999). Communicating this concept to the public proves a challenging proposition, especially considering the fear association with public perception regarding Legionellosis (Irie et al., 2004; Laws et al., 2006).

In Japan, from 1997 to 2000 there was a significant decrease in sales of 24 h hot water baths due the public fear of Legionellosis after the 1996 detection of *L. pneumophila* in a public bath (Irie et al., 2004). In Australia, the largest outbreak of Legionellosis occurred in April 2000 and was caused by the Melbourne aquarium cooling towers. This outbreak resulted in two deaths and 111 identified cases of Legionellosis. The public fear in response to this outbreak was devastating to Melbourne’s tourism, with significant trading losses and legal claims exceeding $35 million (Laws et al., 2006).

One difficulty with communicating risk occurs when a situation is highly publicized and raises significant “public outrage,” for example a cooling tower testing positive for *Legionella.* This causes the potential risk level to be perceived to be much higher than an actual calculated risk level. This is something which must be considered when completing *Legionella* risk assessments as the implementation of risk decisions has a much greater chance of success when supported by the public (Finucane, 2004).

## CONCLUSION

Present regulatory models manage the risk of *Legionella* through strategies maintaining good system health, disinfection residuals and minimizing exposure routes. These regulatory guidelines are informed by *Legionella* risk assessment models which best use the information currently available. The uncertainties associated with each components of *Legionella* risk assessment have been highlighted in this paper. Minimizing these uncertainties will result in improved management protocols. The effectiveness of these management protocols is an important public health issue. Underestimating the risk of *Legionella* may have serious public health consequences; however, overestimating the risk may result in significant economic costs. The paper provides a tool for understanding the uncertainties associated with *Legionella* risk assessment and also provide an overview of the areas that require future research.

## AUTHOR CONTRIBUTIONS

Harriet Whiley authored first draft of manuscript with academic input and expertise provided by Kirstin Ross, Howard Fallowfield, and Alexandra Keegan. All authors were involved in reviewing manuscript and have approved the final version.

## Conflict of Interest Statement

The authors declare that the research was conducted in the absence of any commercial or financial relationships that could be construed as a potential conflict of interest.
